# Utilisation of sexual health services by female sex workers in Nepal

**DOI:** 10.1186/1472-6963-11-79

**Published:** 2011-04-18

**Authors:** Laxmi Ghimire, W Cairns S Smith, Edwin R van Teijlingen

**Affiliations:** 1 Department of Public Health, School of Medicine, University of Aberdeen, Aberdeen, AB25 2ZD, Scotland, UK; 2 Institute of Health Sciences, School of Medicine and Dentistry University of Aberdeen, Aberdeen, AB25 2ZD, Scotland, UK; 3 School of Health and Social Care, Bournemouth University Royal London House Bournemouth, Dorset BH1 3LT, UK

## Abstract

**Background:**

The Nepal Demographic Health Survey (NDHS) in 2006 showed that more than half (56%) of the women with sexually transmitted infections (STIs), including HIV, in Nepal sought sexual health services. There is no such data for female sex workers (FSWs) and the limited studies on this group suggest they do not even use routine health services. This study explores FSWs use of sexual health services and the factors associated with their use and non-use of services.

**Methods:**

This study aimed to explore the factors associated with utilisation of sexual health services by FSWs in the Kathmandu Valley of Nepal, and it used a mixed-method approach consisting of an interviewer administered questionnaire-based survey and in-depth interviews.

**Results:**

The questionnaire survey, completed with 425 FSWs, showed that 90% FSWs self-reported sickness, and (30.8%) reported symptoms of STIs. A quarter (25%) of those reporting STIs had never visited any health facilities especially for sexual health services preferring to use non-governmental clinics (72%), private clinics (50%), hospital (27%) and health centres (13%). Multiple regression analysis showed that separated, married and street- based FSWs were more likely to seek health services from the clinics or hospitals. In- depth interviews with 15 FSWs revealed that FSWs perceived that personal, structural and socio-cultural barriers, such as inappropriate clinic opening hours, discrimination, the judgemental attitude of the service providers, lack of confidentiality, fear of public exposure, and higher fees for the services as barriers to their access and utilisation of sexual health services.

**Conclusion:**

FSWs have limited access to information and to health services, and operate under personal, structural and socio-cultural constraints. The 'education' to change individual behaviour, health worker and community perceptions, as well as the training of the health workers, is necessary.

## Background

HIV is considered as an important STI. The available data on HIV from Nepal indicates that there was a sharp increased in the number of new infections starting in 1996. HIV prevalence among FSWs in Kathmandu Valley was less than 1% in 1995-96 and gradually increased to 2.7% in 1996-97 [1]. The reasons for the increase are attributed to the increased awareness on the part of the FSWs and the general population about HIV and STIs and an increase in the number of service sites. It is estimated that Kathmandu Valley, has 5,000 to 7,000 FSWs, 20% of the total 25,000 to 35,000 FSWs in Nepal [2].

Due to discrimination and stigma and the absence of a systematic recording and reporting system in the health facilities, HIV case reporting is said to be low in Nepal. The reported HIV prevalence among FSWs was 1.8% in 2007 and was reported to be 5.6% in 2009 [3]. The HIV prevalence among the street-based FSWs was reported to have increased to 16% in 1997-98 and 17.1% in 1999-2000 [4]. It declined to 1.4% for both the street-based and establishment-based FSWs in 2004 due to the focused health programme for this target group [5]. The Integrated Biological and Behavioural Surveillance (IBBS) survey in 2009 showed that HIV prevalence among FSWs in Nepal was 2.3% [1]. It is said that a high number of women entered sex work due to the political upheaval, particularly after 2000, and this 'diluting' of the population of FSWs may be the reason for the dramatic fall. The overall prevalence of HIV in the adult population in Nepal is 0.49%, and it is over five percent among injecting drug users (IDUs). The overall prevalence of HIV is a challenge for Nepal to meet the Millennium Development Goal of halting and reversing the prevalence of HIV by 2015.

The syphilis prevalence rates among women in the Beri Zonal Hospital, Nepalgunj and Mahakali Zonal Hospital, Mahendranagar in 2000 were 2% and 1.3% respectively and 4.4% of men had positive results for any STI compared to 7% among the female patients. A similar study done among women by Save the Children US in Kailali District showed 30.6% had sexually transmitted infections (STIs), with an active STI rate of 14%. National data on STIs among Nepali rural women showed a 2.3% prevalence of gonorrhoea, chlamydia and others types of curable STIs [8]. The Nepal Demographic Health Survey (NDHS) in 2006 reported 0.4% women age between 15 to 49 years old self-reported genital discharge and 7.4% women reported sores or ulcers [9]. However, 56% of these women with STIs did not seek any advice or treatment from clinics, hospitals or private doctors or other health workers.

Utilisation of general health services by women, such as antenatal checks (44%) and child birth (19%), is low in Nepal [9]. FSWs do not use routine health services appropriately [10]. There may be barriers on both the supply and demand side of sexual health services FSWs and many factors such as individual, structural and socio-cultural factors for contribute to their low use of sexual health services. One of the reasons for poor rate of treatment-seeking for STIs could be due to the limited number of voluntary counselling and testing centres in Nepal, though they have been increased from 65 in 2004 to 126 in 2008. In Kathmandu, there are 11 voluntary counselling, testing and STI service sites [11].

Stigma attached to sex work is regarded as a barrier to the use of sexual health services and treatment [12]. FSWs are a highly marginalised subgroup [13,14]. The barriers to Kathmandu FSWs using sexual health services and the barriers to accessing health care by them are unknown. There is a lack of information about FSWs own accounts of the sexual health services and their priorities. This study therefore aimed to look at the factors that underline FSW's health seeking behaviours in Kathmandu Valley of Nepal.

## Methods

In this study, utilisation of sexual health services by the FSWs is based on the conceptual framework used by Anderson and Newman [15] for assessing the utilisation of medical care services by women. Therefore this study examined availability, accessibility, affordability, quality of services and individual or internal and service-related factors that are linked to the utilisation of sexual health services.

The study was conducted in 2006 among FSWs aged 17-46 who had been involved in the sex trade in exchanging sex for money for at least six months prior to the survey. We combined a mixed-methods approach in two phases; a questionnaire survey followed by in-depth interviews. We also used a snowball-sampling technique based on a convenience sampling method [16] as FSWs are a hard-to-reach population [17]. We did not calculate a sample size because preparing a sampling frame of FSWs for random sampling and power calculation was not possible. Ethical approval was obtained from the Nepal Health Research Council (NHRC) in order to conduct this study and informed consent was properly taken from the FSWs before their interviews.

We used a pre-tested and structured questionnaire (adopted from FHI/Nepal) and in-depth interview guidelines to collect data. We established contacts with the FSWs through peer educators deployed by the non-governmental organisations (NGOs) as their programmes have reached FSWs in needy areas including in the Kathmandu Valley. A total of 425 FSWs completed the structured interviewer administered questionnaires while a sub-set of 15 FSWs from the questionnaire-based interviews voluntarily took part in the in-depth interviews. Six trained female and two male interviewers conducted questionnaire-based interviews. We involved two male interviewers as some FSWs, during the pilot study, suggested that they were more comfortable telling their personal histories to men rather than women. The first author undertook the in-depth interviews. The in-depth interviews were tape recorded and transcribed in notebooks. They were translated into English and thematically analysed [18]. Quantitative data obtained from the questionnaire survey was analysed by SPSS version 16.

## Results

### Characteristics of the FSWs

A total of 425 FSWs from three cities in Kathmandu Valley participated in an interviewer administered questionnaire survey. The majority of FSWs were interviewed in the cities of Bhaktapur (55.3%) and Kathmandu (41.4%) and only 3% were interviewed in the city of Lalitpur. The ages of the respondents (FSWs) ranged from 15 to 46 years old with a mean age 26 years (Table 1).

**Table 1:** Socio-demographic background of the female sex workers included in the sample (N = 425) in 2006

Variables	N	Percentage
***Age***		
15-19	43	10.1
20-29	269	63.3
30 and above	113	26.6
Mean (SD)	26 (6.04)	
**Level of education**		
Illiterate	144	33.9
Informal-primary level	213	50.1
Secondary and above	68	16.0
**Years of education completed**		
None	275	64.7
≤ 1 year	35	8.2
2-5 years	41	9.6
6-10 years	63	14.8
> 10 years	11	2.6
**Types of FSWs**		
Establishment based	117	27.5
Street based	308	72.5
**Caste/ethnicity**		
Brahmin/Chhetri	154	36.2
Newar	63	14.8
Janajati (Gurung/Rai/Limbu/Magar/Tamang/Sherpa)	187	44.0
Chaudhari/Madhesi	15	3.5
Dalit caste (Kami/Damai/Sarki)	6	1.4
**Religion**		
Hindu	289	68.0
Buddhist	99	23.3
Christian/Muslim	37	8.7

**Total**	**425**	**100**

FSWs are classified into three educational categories- illiterate (those who could not read and write), informal (those women who have education from out of school), primary education (grade 1-5) and secondary (grade 6-12) or above education (Table 1).

The 15 FSWs who participated in the in-depth interviews were aged between 19 and 42 years of age. The interviewees included six married, four separated, four single women and one widowed, three were illiterate, seven literate and five had studied to grades 5-9.

### FSWs general health status

Out of the 425 FSWs who took part in questionnaire-based survey, 380 FSWs reported sickness during the past 12 months and 30.8% reported STI symptoms (see Table 2).

**Table 2:** Distribution of sample female sex workers' self-reported illness in the past 12 months prior to the study (in 2006)

Types of general health problems experienced	N (N = 380) (%)
STIs symptoms	117 (30.8)
Fever	107 (28.2)
Back-ache	93 (24.5)
Headache	63 (16.6)
General cold	52 (13.7)
Diarrhoea	3 (0.8)
Others	9 (2.3)

Percentage total exceeds 100 due to multiple responses

When prompted more than half (58.1%) of the FSWs in the survey reported that they had experienced vaginal discharge or vaginal ulcers in the past 12 months. The proportion experiencing vaginal discharge increased significantly with age (see Table 3).

**Table 3:** Distribution of female sex workers who experienced vaginal discharge in the past 12 months prior to the study (N = 425).

Age group	Vaginal discharge or vaginal ulcer in the past 12 months					
	**Yes**		**No**		**Total**	**Chi-square, P value**
		
	**N**	**Row** **%**	**N**	**Row** **%**	**N**	

15-19	16	37.2	27	62.8	43	
20-29	153	56.9	116	43.1	269	13.42
30 and above	78	69.0	35	31.0	113	P = .001

**Total**	**247**	**58.1**	**178**	**41.9**	**425**	

### Health Service Utilisation for STIs and HIV/AIDS

The questionnaire-based survey showed that 25% of the FSWs had never visited health facilities for the treatment of STIs during their involvement as sex workers and 6% did not visit health facilities thinking that they did not require treatment for STIs. FSWs (21%) had never had a freely available voluntary blood test for HIV. Of the 296 FSWs who reported that they had visited health facilities, 72% visited NGO clinics, 50% private clinics, 27% hospitals and 13% health centres for the treatment of STIs.

### Factors underlying utilisation of sexual health services

FSW's health seeking behaviour and the factors associated with utilisation of health services in this survey cover the four key components; availability; accessibility; affordability and acceptability (or quality of services) [19]. The findings are derived from analysis of the survey and the in-depth interviews.

#### Distance of the clinic

Availability is used to define the range of health care options that are suitable for the needs of the FSWs [19]. The health care options include public hospitals, private clinics, community NGOs, or other health care facilities which the FSWs can utilise when necessary as well as services for the specific use of FSWs such as family planning clinics and STIs clinics.

Female sex workers frequently reported that having local health services was not important for them as they preferred to visit clinics or hospitals in more distant places. They travelled to health facilities far outside their neighbourhoods so that they would not be recognised. FSWs preferred to visit different clinics because sex work is illegal in Nepal and it is culturally unacceptable for unmarried women and widows to purchase condoms. Government hospitals distribute condoms only to married persons and some pharmacies also follow this policy. One FSW shared her experience in this way:

*'I do not like to go to clinics close by, and go to those far away because they (other people) will not recognise me and my work. I also like to change and go to different health facilities for treatment. I do this even when I buy condoms because they (pharmacist) ask me my marital status for condom use.' *(FSW ID 4, age 39)

Another FSW also mentioned that she visited clinics that were far from her community because of fear of exposure of her status to the doctor.

*'I am asked to tell all my problems and my work to the doctor, he will certainly realise that I am a sex worker. Therefore, consulting a doctor in a nearby location is not good for us. It is better to go far away so that no one, neither the doctor nor the patients, has a chance to see and recognise me again.' *(FSW ID 13, age 22)

There was also a fear that, amongst the crowds at government hospitals, there might be someone they know.

*'In the hospitals there are many people lining up for the treatment. I fear that someone could recognise me easily. There may even be a client.' *(FSW ID 1, age 28)

#### Clinic opening hours and waiting time

FSWs consistently reported that they were unable to visit sexual health clinics or hospitals during day time as they had to go to work at the same time, FSWs working in cabin restaurants and on the streets reported that they were busy during day time. At night they go home and are involved in domestic activities. One FSW said this:

*'The government hospital opens only during the day. This is the time we are also busy in our work in cabins or on the streets. We have to find clinics that are open during the evening or morning hours.' *(FSW ID1, age 28)

FSWs complained that they could not afford long waiting times while seeking treatment from the government hospitals. They preferred private clinics because of long waiting times in the government hospitals.

*'Though treatment of sexual health problems was cheaper at the government hospital, we have to be away from work for a full day. In a private clinic we do not have to wait for such a long time.' *(FSW ID 3, age 24)

One FSW ID 6, a sex worker for 13 years, reported her experience visiting Government health facilities for sexual health problems. She had to queue up for three to six hours to get a ticket before seeing a doctor.

#### Affordability

The FSWs frequently raised issues of higher fees charged by the doctors in private clinics alleging that doctors asked for higher fees when they recognised them as sex workers.

*'We are not comfortable with health workers because they charge more money if they recognise us as sex workers.' *(FSW ID 7, age 42)

#### Perceived quality of health services/acceptability

FSWs raised questions about the quality of sexual health services provided by the health facilities

*'There are many private nursing homes (private home) nowadays. I do not believe in their doctors. They are less trained and less experienced than the doctors in the governmental hospitals.' *(FSW ID 1, age 28)

Medical professionals working in private and government hospitals were trained in a common curriculum, but private practitioners may not have had specialised training in STI, as training is provided to the government sector health workers by the Ministry of Health. Another FSW talked about confidentiality being broken:

*'I do not like to tell them (health staff) my personal details and I do not trust their confidentiality. Once my neighbour went with me to the health facility and heard about my work while the doctor was taking my history. Later she asked me about my work, so I had to move from that place.' *(FSW ID 3, age 24)

One FSW (ID 6) said that during registration in a government hospital she had to disclose her symptoms in front of all the other people in the line, which she found humiliating and discriminatory.

FSWs suggested that service providers in government hospitals ask personal questions particularly about their work and sexual history. It was one of the health workers' practices that were disliked by all the FSWs.

*'I visited the hospital last year. The doctor asked me about my past work and when and how I got the disease? When I answered him, he looked at me differently.' *(FSW ID 2, age 19)

FSWs also reported indifference by doctors and other health services providers as a reason for not using government health services. They did not feel comfortable during examination and felt lack of proper care by health service providers.

*'They are not friendly to me. Once they know the nature of my work, they scold me in front of other people. They do not like to talk to me about my problems or issues, and they prescribe medicines without doing proper examinations. The health staffs think that they are doing a good job for the patient, and they have a good reputation. They act like gods and nobody complains about what they are doing.' *(FSW ID 8, age 30)

In Nepal there is no appointment system in place in the hospitals. It is quite normal in a busy hospital or a health clinic that patients are asked several questions before registering them to a particular doctor or unit for treatment.

*'After standing in line for couple of hours, when we reach the ticket window asked loudly our name, age and other details and why we are there. How can we share our problem like that in public?' *(FSW ID 13, age 22)

#### Lack of trust, privacy and confidentiality

Most FSWs interviewed opted to visit private clinics when they needed health care because of greater trust, especially in terms of maintaining privacy and confidentiality. In Nepal, the STI treatment guidelines recommend maintaining the confidentiality of the patients. This is sometimes violated particularly in the case of FSWs. One FSW reported a lack of privacy/shyness as a reason for not visiting the clinics or hospitals thus:-

*'I have not consulted those (clinics) yet because there is no one to help us when we have an urgent need for treatment I am worried about how to tell them that I have these problems (STI)? How can I show my private parts to them? If they ask me about my work, what will I answer?' *(FSW ID 9, age 25)

#### Lack of gender compatibility

A recurrent view was that most health facilities, government and private alike, had male staff and they could not consult them about their sexual health problems. As most of the health service providers in Nepal, particularly doctors providing health services are male, while FSWs preferred female doctors.

*'I will not reveal all my health problems to a male doctor as I am a sex worker and I have sores in my private areas (vulva). I fear he will scold me. When I was visiting a clinic with ulcers once I did not tell the male doctor and he did not ask me my problems either. If a female doctor was available I could tell her.' *(FSW ID 11, age 30)

#### Sexual harassment by the service provider

Some FSWs had experienced sexual harassment by male doctors, though there is a general law in Nepal that forbids sexual harassment of patients by the health professionals. One FSW made a complaint of service provider's prejudice and lack of sensibility like this:

*'In the hospital, the doctor asked me many questions and looked at me differently. He asked me to lie down on the examination table in a room and I did what he instructed. He caught my hand and pressed my body hard. I was scared of him and called my mom who was waiting for me outside. I left the hospital without check- up.' *(FSW ID 3, age 24)

FSWs said that they did not go to NGO clinics due to a lack of knowledge about its sexual health services and for fear of public exposure. As one FSW said this in the following way:

'Many of us do not know where the NGO clinic is located. Even if we do know we are scared of people who know us previously.' (FSW ID 2, age 19)

#### Internal/individual factors

Regression analysis was performed using four demographic factors (age, marital status, education, and FSW type and one attitudinal variable (perceived discrimination). The results of the analysis shows that FSWs above 25 years of age, literate, separated, married and street- based were more likely to seek health services from the clinics or hospitals. However, only marital status and type of sex work were statistically significantly related to FSWs health seeking behaviour. FSWs perceived discrimination by the service providers was not statistically associated with their treatment-seeking behaviours (see Table 4).

**Table 4:** Result of multiple regression analysis on factors affecting FSW's STI treatment- seeking behaviour (N = 296).

Variables	No. (%)	Odds Ratio	95% CI	Significance
**1.Age**				
Below 25	119 (63.9)	1.00	0.830-2.225	0.223
Above 25	177 (74.7)	1.359		
**2. Education**				
Illiterate	98 (68.8)	1.00	0.764-1.890	0.370
Literate	198 (70.5)	1.202		
**3.Marital status**				
Never married	65 (55.6)	1.00		
Separated/divorced/widow	59 (72.8)	2.877	1.756-4.713	0.001
Married	172 (75.8)	2.473	1.320-4.634	0.005
**4. Type of sex workers**				
Establishment-based	71 (60.7)	1.00	1.217-3.117	0.004
Street-based	83 (73.1)	1.948		
**5. Perceived health worker discrimination in**				
Yes	142 (67.6)	1.00	0.540-1.284	0.425
No	154 (71.6)	0.833		

OR = Odds Ratio, CI = Confidence Interval

## Discussion

FSWs participating in this study were from diverse backgrounds in terms of age, education, marital status, caste, ethnicity and work settings. Nearly one-third self-reported that they had suffered from symptoms of STIs, particularly vaginal discharge, itching around the genitals, sores and fever. The survey findings show that more than a quarter had never visited any health facilities seeking for health care services. Moreover, one-fifth of the survey respondents had not had voluntary blood testing for HIV which is available locally free of cost. The majority who sought health services turned to NGO clinics, private clinics and government health facilities. In-depth interview participants frequently mentioned that they had visited private clinics and pharmacies as well as government health facilities.

The findings suggest a number of structural or external and individual or internal factors tend to serve as enablers or barriers for FSWs use of sexual health services. The structural factors include service-related problems such as the distance of the service sites, clinic opening hours and waiting times, service fees, perceived quality of the service, particularly with regard to training and the quality of service providers, a lack of confidentiality and privacy, disrespectful and judgemental attitudes of the service providers, the presence of male service providers, a lack of trust and fear of stigma and embarrassment by the service providers. These findings are similar to a previous study by Tandukar in 2003 [20] which attributes women's' low utilisation of STI related health services to insufficient knowledge of STIs, fear, stigmatisation, women's secondary status, and the presence of male health workers in the health institutions.

FSWs prefer distant health clinics because of social stigma and fear of exposure to the public. Having a sexual health clinic near the location of their work does not appear to help increase FSWs utilisation of health services. FSWs perceived that the quality of doctors was higher in the government hospitals, but they lacked privacy and confidentiality, were inaccessible outside daytime hours, and was characterised by long waiting hours. FSWs were concerned with private clinics and hospitals for charging higher fees. There is a lack of government regulation about the service charges of private health services. Moreover, FSWs perceived that doctors and nurses, particularly in the private clinics, were poorly trained and less experienced in STIs. This finding supports previous studies [21,22].

The findings from regression analysis suggest internal factors including age, education, work setting, marital status and perceived discrimination tend to be key factors determining the utilisation of sexual health services by FSWs. FSWs above 25 years of age, literate, separated, married and street- based FSWs were more likely to seek health services from the clinics or hospitals. However, only marital status and type of sex work were statistically significantly related to FSWs health seeking behaviour. FSWs perceived discrimination by the service providers was not statistically significantly associated with their use of health services. These findings suggest the need for targeted interventions for the younger, unmarried and establishment-based female sex workers in addition to the general interventions on FSWs.

The structural and internal factors identified are similar to previous studies conducted elsewhere [23,24]. They classify the underlying factors in different ways; individual and structural factors or internal and external factors and they recognised that obtaining sexual health services was a stressful experience fraught with both internal and external barriers. As reported, these factors limit availability, accessibility, affordability and the quality of the services to be provided to FSWs. Their vulnerability is further increased due to sex work being illegal in Nepal [25-27] though it is legal in Brazil, New Zealand, Rhode Island (US), Queensland (Australia), Canada and several European countries [28,29]. Interventions comprising education, the training of health workers on interpersonal communication, flexible clinic opening hours, and the reduction in stigma for empowering FSWs could improve their use of existing health services.

## Study Limitations

The findings presented in this paper are based on self-reporting by the FSWs from within the Kathmandu Valley. As sex work is illegal in Nepal, FSWs may have provided some inaccurate or incomplete information. It was not possible to select participants randomly because there were difficulties in identifying a sampling frame of FSWs and secure venues where interviews could be conducted due to the fear of media exposure and previous episodes of confidentiality violations. FSWs are also highly mobile due to the fear of police and other armed personnel. The sensitive nature of questions sometimes generated emotional responses in the FSWs and it was often difficult to complete interviews when discussing painful experiences. This study did not include the service providers' perspectives and observation of the response of the service providers to the FSWs.

## Conclusion

Several structural, internal or individual and cultural factors determine the use of health services by FSW such as availability, accessibility, affordability and perception regarding the quality of sexual health services. FSW's perceived a lack of privacy and confidentiality at government health facilities created distrust among FSWs and higher fees for services at private clinics prevented the use of sexual health services. Social stigma attached with fear of exposure as a sex worker and health worker discrimination and judgemental behaviours appear to be the major barriers to seeking health services.

## Abbreviation used

(FSWs): STI/AIDS Counselling and Training Service (SACTS) is a non-profit non-governmental organisation. Female sex workers; (New ERA): New ERA is one of the first non-government, non- profit research organization in Nepal; (FHI): Family Health International; (NCASC): National Centre for AIDS and STD Control; (STI): Sexually Transmitted Infections.

## Competing interests

The authors declare that they have no competing interests.

## Authors' contributions

LG collected and analysed data and drafted the manuscript. WCSS redrafted the manuscript and a substantial contribution to the design of the study. ERvT supervised the data analysis, design of the study and redrafted the manuscript and all authors have read and approved the final version.

## Author's information

At the time of this study, LG was a PhD student in the School of Medicine, Public Health at the University of Aberdeen. WCSS is professor and head of department in population health at University of Aberdeen. ERvT is professor Centre for Midwifery and Maternal & Prenatal Health, School of Health & Social Care at Bournemouth University.

## Pre-publication history

The pre-publication history for this paper can be accessed here:

http://www.biomedcentral.com/1472-6963/11/79/prepub
